# HIV-1C proviral DNA for detection of drug resistance mutations

**DOI:** 10.1371/journal.pone.0205119

**Published:** 2018-10-04

**Authors:** Kahsay Huruy, Andargachew Mulu, Uwe Gerd Liebert, Maier Melanie

**Affiliations:** 1 Department of Medical Microbiology, School of Biomedical and Laboratory Sciences, University of Gondar, Gondar, Ethiopia; 2 Armauer Hansen Research Institute (AHRI), Addis Ababa, Ethiopia; 3 Institute of Virology, Leipzig University, Leipzig, Germany; National and Kapodistrian University of Athens, GREECE

## Abstract

**Background:**

Using HIV proviral DNA as a template may be suitable for initial detection of transmitted drug resistance mutations (TDRMs) as it is easy to handle and less expensive compared to RNA. However, existing literatures which are mainly focused on HIV-1B subtypes DNA extracted from PBMCs revealed controversial findings ranging from the detection of significantly lower or higher mutations in proviral DNA compared to historic viral RNA. Thus, to verify whether viral RNA or proviral DNA has improved sensitivity in detecting transmitted genotypic drug resistance mutations paired viral RNA and proviral DNA (which is directly extracted from stored whole blood) samples were tested from Ethiopian antiretroviral naive HIV-1C infected subjects.

**Methods:**

In the present comparative study the frequency of TDR mutations was assessed in paired samples of viral RNA and proviral DNA (extracted directly from stored whole blood) of HIV-1C infected treatment naïve patients and interpreted using the 2009 WHO drug resistance surveillance mutation lists, Stanford University drug resistance data base and International Antiviral Society-USA mutation lists.

**Results:**

High agreement in rate of TDR between the two compartments was observed using the WHO mutation lists. While mutations G190A and E138A were concurrently found in both compartments, others such as G73S on PR and A62V, M184I, M230I on RT were identified in proviral DNA only. All signature mutations seen in viral RNA were not missed in proviral DNA.

**Conclusions:**

The concordance of major genotype drug resistance mutation between RNA and proviral DNA in treatment naïve patients suggests that proviral DNA might be an alternative approaches for an initial assessment of drug resistance prior to initiation of antiretroviral therapy using the WHO mutations lists in resource-limited countries. However, the clinical importance of TDRMs observed only in proviral DNA in terms of being a risk factor for virologic failure and whether they limit future treatment options needs additional investigation using more sensitive sequencing approaches such as Next Generation Sequencing (NGS).

## Background

Since the introduction of antiretroviral therapy (ART), the relative rates of morbidity and mortality from acquired immunodeficiency syndrome have been reduced significantly [1]. However, the clinical benefits of ART are jeopardized by the emergence of drug-resistant HIV-1 strains [2, 3]. The currently available ART is directed against virus-specific enzymes as well as the processes of attachment and entry into cells and are not curative nor eradicate HIV infection, as latent infection is established through the integration of double-stranded proviral DNA into the host cell which is essential during HIV life cycle [4]. The proviral DNA archives viral variants and preserves them until transcription and translation is initiated in these cells again. These cells, at least temporarily not producing virus, represent a barrier to viral eradication and it is inherently difficult to specifically target and remove such cells [5]. Accordingly, proviral DNA has been investigated as an additional source of information on HIV-1 resistance analysis [6–11]. Simultaneous analysis of viral RNA and proviral DNA apparently increases the sensitivity of TDR detection and testing of proviral DNA may also be important for monitoring treatment outcomes [12]. However, existing literatures which are mainly focused on HIV-1B subtypes revealed controversial findings ranging from the detection of significantly lower [6, 8] or higher [9, 11] mutations in proviral DNA compared to historic viral RNA to a high level of genotype concordance between RNA and proviral DNA in newly diagnosed viremic patients [13–15] and in a viremic patients under ART [13, 15]. Moreover, most of the above comparative studies were conducted on DNA extracted from PBMCs which is more labor intensive and expensive to prepare and requires at least -70°C cold storage which is not available in most laboratories of the developing countries.

Thus, to verify whether viral RNA or proviral DNA has improved sensitivity in detecting transmitted genotypic drug resistance mutations paired viral RNA and proviral DNA (which is directly extracted from stored whole blood) samples were tested from antiretroviral naive HIV-1C infected subjects from Ethiopian where HIV-1 transmitted drug resistance is increasing [16–21].

## Materials and methods

Four ml of whole blood was collected in EDTA-containing tubes from 41 ART naïve HIV-1C infected patients from Northwest Ethiopia (Gondar University and Metema Hospitals) during January to March 2010. For preservation of cells, blood samples were immediately mixed with ethylene and propylene glycol (EPG, Sigma Chemical Co., Gaithersburg, Md, USA; final EPG concentration 20%) [22] and stored at -20 °C. CD4^+^ T-cell count was performed on fresh whole blood by flow cytometry (FACScount system, Becton Dickinson, San Jose, CA, USA) following the manufacturer’s specifications. Details of patient’s recruitment and management has been described elsewhere [20].

Extraction of plasma RNA, HIV-1 viral load determination and HIV-1 viral genome amplification and sequencing of protease codons 1–99 and reverse transcriptase codons 1–331 was done as described previously [17]. DNA was manually extracted from the cellular fraction according to published protocol with minor modifications [23]. Briefly, PBS (phosphate buffered saline) containing 0.1% NP-40 was added at a ratio of 1:5 to 1 ml of whole blood supplemented with EPG and centrifuged at 3,000 x g for 5 min. The pellet was re-suspended in PBS (1 ml containing 5% SDS) and incubated at 37 °C for 30 min. Thereafter, equal volume of lyses buffer (8 M urea, 0.3 M NaCl, 10 mM EDTA, 10 mMTris-HCl pH 7.5) was added, followed by further incubation at 37 °C for 30 min. Total DNA was manually extracted by phase extraction using organic solvent, followed by ethanol precipitation.

Amplification of HIV-1 proviral DNA was done using the same protocol as for viral RNA [17] but omitting the RT step. PCR final products for viral RNA and proviral DNA were purified using Wizard SV Gel and PCR Clean-up kit (Promega, Madison, WI, USA). The purified PCR products were sequenced using the fluorescent dideoxy-terminator method and the ABI Prism 3100 genetic analyser (Applied Biosystem, Foster City, CA, USA). FASTA files were analysed using Stanford University HIV-1 Sequence Database (http://hivdb.stanford.edu), which contains a computer assisted interpretation of mutational profiles providing five levels of inferred drug resistance. All except the fully susceptible HIV-1 sequences were considered as resistant in the analysis of the present study. Each sequence from both compartments was also analysed using WHO mutation lists (http://cpr.stanford.edu.cpr.cgi) and the updated 2017 International Antiviral Society (IAS) mutation lists [24]. All sequences obtained from both, viral RNA and proviral DNA, were confirmed using the Rega HIV-1 subtyping tool, version 2.0 (http://www.bioafrica.net/subtypetool). Phylogenetic tree was constructed by the neighbour-joining method using data generated from the *pol* region (RT and PR genes) of all viral RNA and proviral DNA sequences. The reliability of the branching orders was assessed using the bootstrap approach with MEGA version 5 [25]. Seventeen reference strains, 9 representing HIV-1C and 8 representing non-B subtypes were included for comparison. The nucleotide sequences were deposited at the National Centre for Biotechnology Information (NCBI), USA GenBank (accession numbers KJ807732 to KJ807772 for proviral DNA and KJ807649 to KJ807689 for viral RNA).

Statistical analysis was carried out using SPSS software, version 17.0 (SPSS, Chicago, II., USA). Mean and standard deviation (SD) were computed for the different variables. A *P*-value less than 0.05 was considered statistically significant and 95% confidence intervals (CI) were computed by GraphPad Prism software version 5 (San Diego, CA, USA).

## Ethics approval and consent to participate

All of the protocols using human specimens including written consent were approved by The University of Gondar Ethical Review Committee (RPO 55/258/2001) and written consent was obtained from all of the subjects.

## Results

The characteristics of the study subjects (n = 41) are summarized in Table 1. Thirty-three were in age group 26–45 years and 24 (58.5%) were men. Only eight patients (19.5%) had more than 200 CD4^+^ T cells per ml. The mean ± SD of viral load (log_10_ copies/ml) and hemoglobin values (g/dl) of the patients was 4.76 ± 0.60 and 10.98 ± 2.5, respectively. Female gender but not age, baseline CD4 cell count and hemoglobin values was significantly associated with drug resistance [odds ratio 6.22, p = 0.020].

**Table 1:** Baseline characteristics of patients.

Total numberofpatients	41
Sex	
Men	24 (58.5%)
Women	17 (41.5%)
Age (years)	
21–25	5 (12.2%)
26–35	23 (56.1%)
36–45	10 (24.4%)
≥ 46	3 (7.3%)
Mean ± SD of hemoglobin (g/dl)	10.98 ± 2.5
CD4 cell count (cells per mm^3^)	
< 50	11 (26.8%)
50–99	11 (26.8%)
100–199	11 (26.8%)
200–320	8 (19.5%)
Mean ± SD of HIV RNA viral load (log_10_ copies per ml)	4.76 ± 0.60

**Fig 1:**
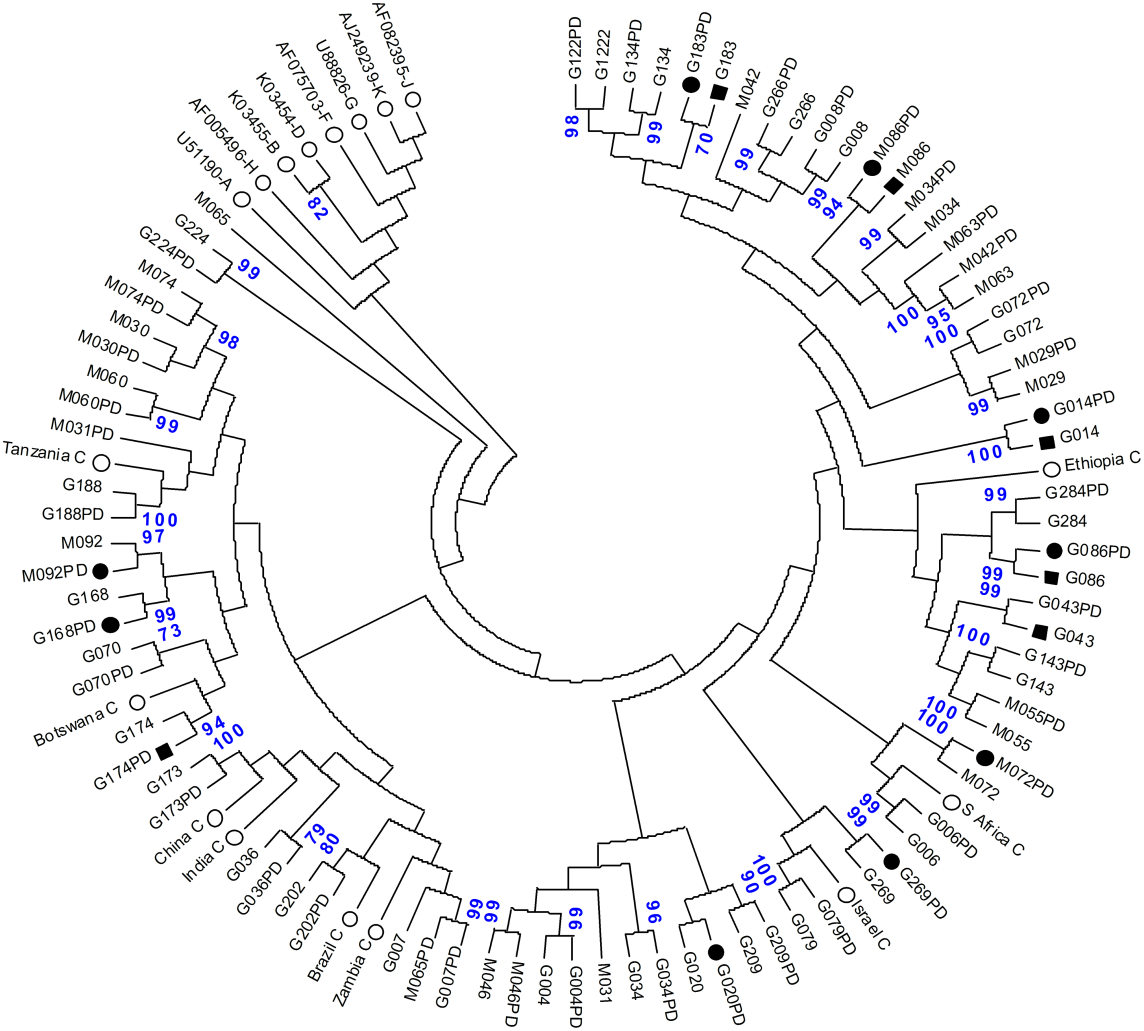
The neighbour-joining phylogenetic tree depicting the relationship between viral RNA (indicated with letter and number) and proviral DNA (indicated with letter, number and PD) from 41 treatment naïve patients in Northwest Ethiopia. Patients who developed HIV-1 drug resistance (black circles for HIV-1 proviral DNA and black rectangles for HIV-1 RNA) also indicated. For comparison, 17 reference strains (empty circles) from the Los Alamos HIV-1 database representing subtype C with GenBank accession numbers U46016 (Ethiopia C), AF067155 (India C), AF110980 (Botswana C), AF361874 (Tanzania C), AY772699 (S Africa C), U52953 (Brazil C), AF286233 (Israel C), AF286230 (China C), AF286225 (Zambia C), and A,B,D,F,G,H,J,K subtypes are included. The nucleotide sequences were submitted to GenBank (accession number: KJ807732—KJ807772 and KJ807649—KJ807689 for proviral DNA and viral RNA, respectively).

The *pol* consensus sequences consisting of 1,261 nucleotides were analyzed to assess the similarity between the viral RNA and proviral DNA (Fig 1). For all patients the infecting virus was identified as HIV-1C. The phylogenetic tree reveals a high genetic similarity between viral RNA and proviral DNA. However, in four paired samples (G7, M31, M42, and M65) RNA and DNA sequences clustered at different branches.

Overall, sequence profiles of both RNA and proviral DNA were concordant in 83% (34/41) of the patients. Thirty patients had wild type genotype in both PR and RT region of the pol genome from both viral RNA and proviral DNA; eleven patients (26.8%, Table 2) had a mutant type from both compartments conferring transmitted drug resistance, of which in four patients mutations were concurrently detected both in RNA and proviral DNA; in two patients mutations were only found in viral RNA (patients ID G043 and G174) and in five patients drug resistance mutations occurred only in proviral DNA (patients ID G020, G168, G269, M072 and M092). Despite differences between the different algorithms, in all algorithms DNA is more sensitive to detect drug resistance mutations and none of the signature mutations found in viral RNA was missed in proviral DNA. High agreement in rate of TDR between HIV RNA and proviral DNA was observed using the WHO drug resistance surveillance mutation lists with coefficient of agreement (Table 3). The prevalence of TDR is summarized in Table 2. The overall rate of TDR to at least one drug class using WHO mutation lists, Stanford University Algorithm database and the IAS mutation lists was 19.5% (8/41 patients, CI 10.0–34.3%), 22.0% (9/41, CI 11.8–36.9%) and 26.8% (11/41; CI 15.6–42.1%), respectively (Table 3). Out of the 5 patients with drug resistant associated mutations in proviral DNA, three had ≥2 APOBEC3G/F signature mutations (M184I and M230I) in reverse transcriptase region with G-to-A mutations. However, since we do not know the role of of APOBEC3G/F activity on future virological outcomes (particlualry in HIV-1C isolates), we prefered to count these mutations in the rate of TDR.

**Table 2:** Characteristics of patients with transmitted drug resistant strains.

Code	Age (years)	Gender	CD4^+^ count (cells/mm^3^)	Viral load (log_10_ copies/ml)	Drug resistance associated mutations					
					PI		NRTI		NNRTI	
					Viral RNA	Proviral DNA	Viral RNA	Proviral DNA	Viral RNA	Proviral DNA
G014	35	Female	253	5.65	-	-	-	-	G190A*^+^^#^	G190A
G020	31	Female	104	4.34	-	G73S*^+^	-	-	-	M230I^#^
G043	44	Female	62	4.31	M46I*^+^^#^	-	-	-	-	-
G086	55	Female	36	4.82	-	-	-	-	E138A^#^	E138A
G168	30	Female	75	4.92	-	M46I	-	-	-	M230I
G174	30	Femae	34	4.52	-	-	-	-	G190E*^+^	-
G183	27	Female	29	4.49	-	-	-	-	E138G^+^^#^	E138AG^+^^#^
G269	35	Male	20	5.58	-	-	-	A62V^#^	-	-
M072	26	Female	18	4.94	-	-	-	M184I*^+^^#^	-	M230I
M086	28	Male	201	4.11	-	-	-	-	G190A	G190A
M092	30	Male	91	4.41	-	-	-	M184I	-	M230I

PI-protease inhibitor; NRTI-nucleotide reverse transcriptase inhibitor; NNRTI-non- nucleotide reverse transcriptase inhibitor; (-) no drug resistant mutations detected;

^(*)^ mutations found in WHO mutation lists;

^(+)^ mutations according to Stanford University drug resistance database;

^(#)^ mutations found in IAS lists.

**Table 3:** TDR frequency according to different algorithms.

	Number of patients (relative frequency)		
TDR found in	WHO	Stanford	IAS
Viral RNA	4 (9.8%)	5 (12.2%)	5 (12.2%)
Proviral DNA	6 (14.6%)	7 (17.1%)	9 (22.0%)
Overall TDR	8 (19.5%)	9 (22.0%)	11 (26.8%)

Four out of eleven patients (G20, G168, M72 and M92) had two TDR in their proviral DNA representing multi-drug resistance (Table 2). The rest seven patients (17.1%) harboured a virus with one mutation each either in viral RNA or proviral DNA or both compartments. The profile of TDR was: in four patients mutations (i.e. G190A, E138A/G) were found concurrently both in viral RNA and proviral DNA, in two patients resistance mutations (M46I and G190E) were detected only in viral RNA, and in five patients archived transmitted drug resistance mutations (M230I, G73S, M184I, M46I, and A62V) in proviral DNA were identified. TDR appeared to be higher in patients with CD4^+^ cell count < 100 cells/mm^3^ (72.7%) and in patients who were in the age group of 26–35 years (81.8%, Table 2). However, these differences were not statistically significant (*P*> 0.05). TDR to NNRTI was identified in 22.0% (9/41) of patients from both, viral RNA and proviral DNA, and in three patient’s resistance mutations to PIs. NRTIs related mutations M184I and A62V were only found in proviral DNA (Table 2). HIV-1 proviral DNA from sample G168 had two archived transmitted drug resistance mutations (M46I and M230I) the same is true for G020 (G73S and M230I). Please note that, E138A is a polymorphic change depending on subtype and could occur in 0.5% to 5% of viruses from treatment naive patients. However, its presence reduces ETR and RPV susceptibility by about 2-fold and hence the presence of E138A prior to therapy may reduce the antiviral activity of RPV. Thus, E138A is considered as a TDR mutation.

The abundance of polymorphisms (Table 4) at known drug resistance positions were significantly higher in proviral DNA (24.4% (10/41); 95% CI 13.7–29.5%) than in viral RNA (17.1% (7/41); 95% CI, 8.2–31.6%). Polymorphic changes at the drug resistance sites were more frequent in PR region than in the RT region in both compartments where 80% (8/10) and 85.7% (6/7) were identified in plasma RNA and proviral DNA, respectively. Positions V82I (PR) and A98S (RT) were most common polymorphic alterations in both compartments (Table 5).

**Table 4:** Occurrence of polymorphisms at drug resistance sites.

	Antiretroviral drug class			
	PI	NRTI	NNRTI	Sum
Viral RNA	1 (2.4%)	2 (4.9%)	4 (9.8%)	7 (17.1%)
Proviral DNA	2 (4.9%)	3 (7.3%)	5 (12.2%)	10 (24.4%)

**Table 5:** Frequency of polymorphisms at drug resistance positions in HIV viral RNA and proviral DNA in treatment naïve patients.

Location	PI mutations		Location	NRTI mutations		Location	NNRTI mutations	
PR	Viral RNA	Proviral DNA	RT	Viral RNA	Proviral DNA	RT	Viral RNA	Proviral DNA
L23	-	-	M41	1(I)	1(I)	A98	7(S)	8(S)
L24	-	-	A62	-	-	L100	-	-
D30	-	-	K65	-	-	K101	1(R)	1(R)
V32	-	-	D67	-	-	K103	-	-
L33	-	-	T69	-	1(S)	V106	-	-
M46	-	-	K70	-	-	V108	-	-
I47	-	-	L74	-	-	V179	2(I)	3(I)
G48	-	2(R)*	V75	-	-	Y181	1(F)	-
I50	-	-	F77	-	-	Y188	-	-
F53	-	-	Y115	-	-	G190	-	2(R)
I54	-	-	F116	-	-	P225	-	-
G73	-	-	V118	1(I)	1(I)	F227	-	-
L76	-	-	Q151	-	-	M230	-	-
V82	5(I)	5(I)	M184	-	-	E138	-	-
I84	-	-	L210	-	-	P236	-	1(PT)
N88	-	-	T215	-	-			
L90	-	-	K219	-	-			

PR-protease; RT-reverse transcriptase; NRTI-nucleoside reverse transcriptase inhibitor; NNRTI- non-NRTI; (-) no polymorphisms detected;

^(*)^-number of mutation (mutant amino acid)

## Discussion

In the present study, 83% (34/41) of the patient’s had concordant sequence profile in determining transmitted drug resistance from both RNA and proviral DNA and those major mutations conferring transmitted drug resistance in RNA (the gold-standard template for HIV drug resistance testing as it reflects the virus that is actively reproducing) were also concordantly detected in proviral DNA consistent with recent finding [13] and suggest that proviral DNA could be used as an alternative template for determining transmitted drug resistance particularly in developing countries considering the fact that: i) RNA genotyping requires RNA extraction and storage at least at -70°C which in most cases is not available in developing countries; ii) RNA needs reverse-transcription which is costly and RNA could easily be degraded before reverse-transcription, impairing amplification and sequencing quality. On the other hand, unlike other studies which used PBMCs, this study used whole blood preserved in 20% EG and stored at -20°C, followed by DNA extraction subjected to direct amplification and sequencing shows the durability and long term stability of whole blood proviral DNA and thus using of proviral DNA directly from whole blood as a template for determining transmitted drug resistance is less expensive. However, there was difference between the RNA and proviral DNA in drug resistance associated mutations for NRTIs (0 vs 3 samples) which could represent archival genetic resistance which may be transmitted or re-emerge with treatment [14].

The inconsistencies between plasma viral RNA and peripheral blood mononuclear cell proviral DNA in HIV-1 drug resistant strains have been reported from treatment naive individuals ranging from the detection of significantly lower [6, 8] or higher [9, 11] mutations in proviral DNA compared to historic viral RNA. Although, the clinical relevance of transmitted drug resistance mutation in proviral DNA among these treatment naïve patients is not defined in the current study, they may not affect the clinical out-comes of ART in short period of time [26]. However, whether these transmitted drug resistance mutation in proviral DNA will be definite risk factors for virologic failure and therefore limiting future treatment options in the course of infection with HAART needs additional investigation. Indeed, it has been shown that the long term persistence of archived drug resistant HIV-1 proviral DNA could undermine the capability of targeted treatment(s) and represents the potential resistance pattern in future [3, 27].

The observed lower abundance of TDR in viral RNA than proviral DNA (14.6% vs 22%) could be conceivably a result of the reversion over time of HIV-1 quasispecies resistant to HAART [28] being archived in proviral DNA [6, 9, 11]. The occurrence of hypermutation in 3 out of 5 sequence of the reverse transcriptase region of the proviral DNA is consistent with recent studies [29].

Four out of the 41 paired samples from both compartments did not show a tight proximity relation to each other suggesting that circulating strains produced in the plasma compartment could arise from sources other than circulating infected leukocytes [14, 30]. Most of the studies on the comparison of HIV-1 drug resistance in cell-free and cell-containing compartments were undertaken in HAART treated subjects [10, 14, 30–33]. The available few data among treatment naïve patients [6, 8, 9, 11] were exclusively conducted in HIV-1B isolates. To the best of our knowledge this is the first report among HIV-1C isolates which compares the profile of transmitted drug resistance mutation from paired RNA and proviral DNA templates. The 22.0% of TDR to NNRTIs matches well with earlier observations where NNRTI associated drug resistance mutations were found more frequently than mutations against other ART classes both in HIV-1B [34]and non B subtypes [35, 36]. The profiles of TDR mutations between viral RNA and proviral DNA in the present study are variable in such a way that TDRs mutations conferring resistance to RTIs and PIs were identified more frequently in proviral DNA than viral RNA consistent with previous studies among treatment naïve patients [6, 8, 37]. In contrast to these findings, several other studies showed comparable prevalence of TDRs from RNA and proviral DNA among treatment naïve patients [38, 39] and ART experienced and failing patients with history of TDR [10, 11, 27].

## Limitations

The data has an obvious limitations including small sample size with few detected drug resistance mutation. Although there is technical improvements in the amplifcation of drug resiatnce genome using direct whole blood as a template, the technical improvment over sequencing is limitted. It would have been interesting to evaluate the use of more sensitive sequencing approaches such as Next Generation Sequencing (NGS) that might partially explain the observed differences between viral DNA and plasma RNA. Thus, the observd small mutation might be due to the low sensitivity nature of of the Sanger sequencing approach. Furthermore, as the nature the study was cross sectional, the clinical importance of the mutations observed only in proviral DNA in terms of being risk factors for virologic failure and whether limits future treatment options is not addressed. Nevertheless, with all these limitation we concluded the points below (Conclusions).

## Conclusion

In summary, as sequencing of HIV-1 proviral DNA which is extracted directly from stored whole blood detects all signature mutations observed in RNA and is less expensive and technically easier to perform compared to RNA, determining TDR using the WHO drug resistance surveillance mutation list from proviral DNA could be an alternative approaches especially in resource limited countries. However, the clinical importance of transmitted drug resistance mutations observed only in proviral DNA in terms of being risk factors for virologic failure and limits future treatment options in future needs additional investigation using more sensitive sequencing approaches such as Next Generation Sequencing (NGS).
