# Correction: HIV-1C proviral DNA for detection of drug resistance mutations

**DOI:** 10.1371/journal.pone.0207079

**Published:** 2018-11-01

**Authors:** Kahsay Huruy, Andargachew Mulu, Uwe Gerd Liebert, Melanie Maier

The fourth author’s name is spelled incorrectly. The correct name is: Melanie Maier. The correct citation is: Huruy K, Mulu A, Liebert UG, Maier M (2018) HIV-1C proviral DNA for detection of drug resistance mutations. PLoS ONE 13(10): e0205119. https://doi.org/10.1371/journal.pone.0205119
